# Optimizing influenza vaccine policies for controlling 2009-like pandemics and regular outbreaks

**DOI:** 10.7717/peerj.6340

**Published:** 2019-01-28

**Authors:** Sheng-I Chen, Chia-Yuan Wu, Yu-Hsuan Wu, Min-Wei Hsieh

**Affiliations:** Department of Industrial Engineering and Management, National Chiao Tung University, Hsinchu, Taiwan

**Keywords:** Disease model, Influenza, Vaccine policy, Simulation

## Abstract

**Background:**

This study examined the effectiveness of various vaccine policies against influenza. The transmission rate was calculated by use of the time-series influenza-like illness case during the year of 2009 and recent epidemics in Taiwan.

**Methods:**

We developed a stochastic compartmental model to analyze the transmission of influenza, where the population was stratified by location and age group, and the vaccine distribution was considered using the current policy. The simulation study compared the previous vaccine policy and a new policy with expanded coverage and various lengths of the vaccination campaign. The sensitivity analysis investigated different levels of vaccine efficacy to confirm the robustness of the recommended policies.

**Results:**

Doubling vaccine coverage can decrease the number of infections effectively in the regular epidemic scenario. However, a peak of infections occurs if the duration of implementing vaccination is too long. In the 2009-like pandemic scenario, both increasing vaccine doses and reducing the program’s duration can mitigate infections, although the early outbreak restricts the effectiveness of vaccination programs.

**Conclusions:**

The finding indicates that only increasing vaccine coverage can reduce influenza infections. To avoid the peak of infections, it is also necessary to execute the vaccination activity immediately. Vaccine efficacy significantly impacts the vaccination policy’s performance. When vaccine efficacy is low, neither increasing vaccination doses nor reducing vaccination timeframe prevents infections. Therefore, the variation in vaccine efficacy should be taken into account when making immunization policies against influenza.

## Introduction

**Figure 1:**
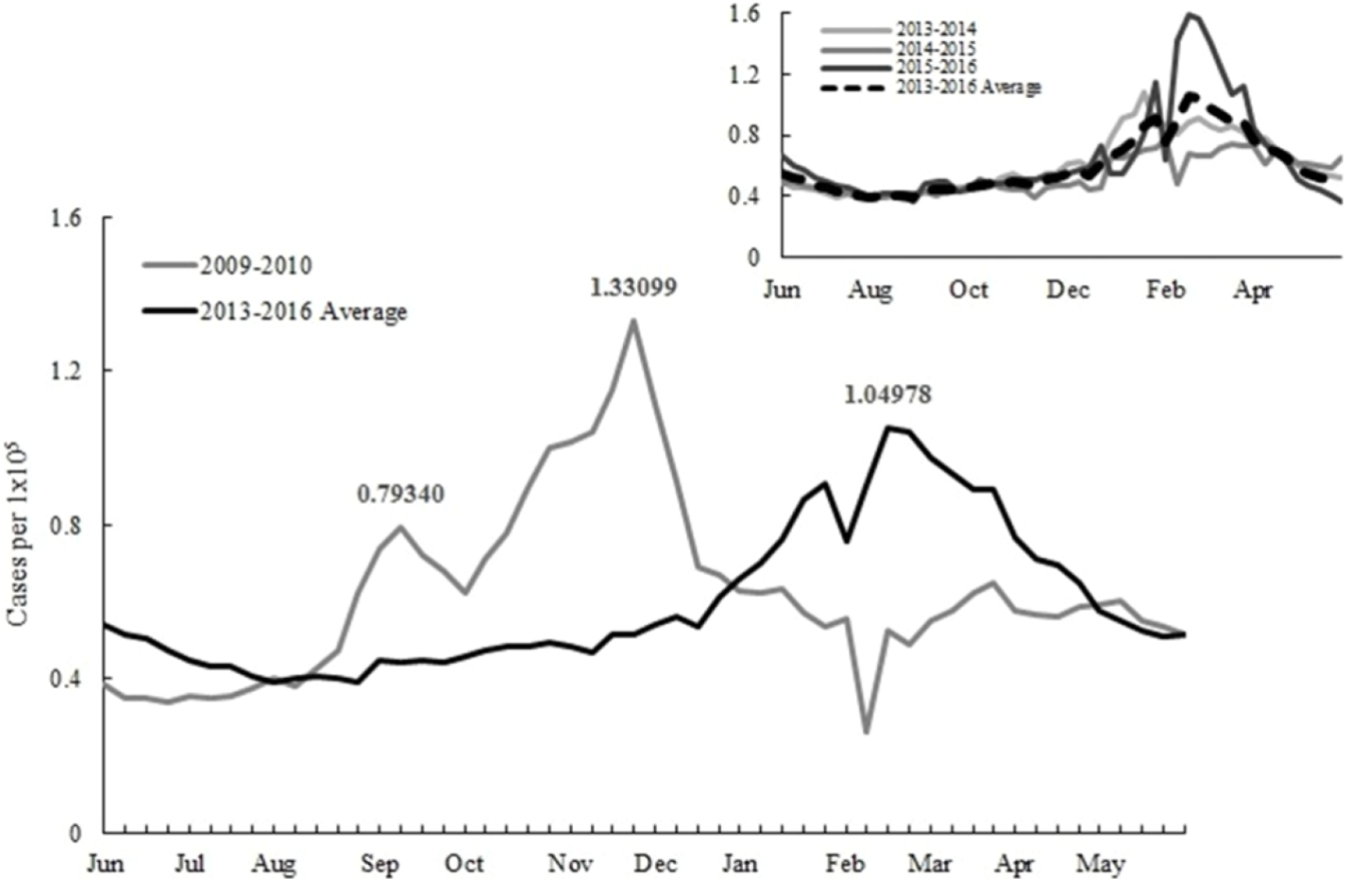
Reported influenza-like illnesses during the 2009 and 2013–2015 seasons.

The influenza vaccination starts at the beginning of October for most countries in the northern hemisphere to protect people from infections. Irregular epidemics have posed a challenge for implementing such a fixed vaccination schedule. The objective of this study is to examine vaccination policies under various epidemic scenarios and provide suggestions for preparing future epidemics. A relevant example is a pandemic in 2009 when new influenza A H1N1 virus emerged and rapidly caused global infections (Chuang et al., 2012). The epidemic pattern in 2009 is distinct from other years. There were double outbreaks in 2009 in Taiwan. The first infection peak in September caused 0.79 ILI cases per 100,000 citizens and then followed by another transmission with a larger magnitude in the late November (Fig. 1).

The shifting viral strain is one of the key factors associated with the pandemic. After observed a number of infections in spring 2009, a new H1N1 virus had been identified. The viral strain had been spread globally, but it was not included in the regular seasonal vaccine. Because the production of the seasonal vaccine already started, vaccine manufacturers were unable to alter vaccine products to include the new viral strain. The influenza-like illness (ILI) defines as the acute respiratory infection with the symptom of a cough and a fever of ≥ 38 C°(World Health Organization, 2018). There were a large number of clinical visits for ILI before the monovalent vaccine was available in early November. The H1N1 outbreak continued the following year, and there were sporadic infections during the post-pandemic period (Chuang et al., 2012; Centers for Disease Control, 2017b). Although the new H1N1 virus caused a large size outbreak in 2009, the epidemic caused less mortality than an average season due to many infections occurred in the younger population. Therefore, the World Health Organization (WHO) defined the pandemic as a moderate severity level (World Health Organization, 2009).

After the first outbreak in September 2009, the Taiwanese government announced a quarantine policy (known as the 325 policy) on campuses to protect school-aged children from infections. This policy ensures that the school must be shut down for five days if there were two new infected cases within three days. As the pandemic vaccine was available in November, the quarantine policy was canceled by the government if the school achieved an 80% vaccination rate. The vaccinated population was about 5.6 million during the flu season, and approximately 520,000 individuals received vaccines on the national vaccination day, December 12. Besides using vaccines, the government also ordered 60 million masks for controlling the outbreak (Centers for Disease Control, 2017b). The overall vaccine rate was at an undesirable level in 2009, although the government has allocated abundant resources to control the disease. There were millions of leftover vaccines at the end of the vaccination program, and only one-third of vaccine orders were applied to the population. The low vaccination rate mainly associated with vaccination side effects, such as dizziness and vomiting that occasionally occurred in the younger population (Centers for Disease Control, 2018). Not just in Taiwan but also other countries noted adverse cases after receiving the pandemic vaccine. Concerns regarding the monovalent vaccine side effect have been discussed in the literature, where the author suggests the information of disease background and vaccination benefits should be available to the public in a transparent and easy-to-understand manner (Poland, 2010).

The economic benefit of vaccination has confirmed by various studies using modeling approaches (Maciosek et al., 2006). Examples of studies investigated the economic benefits of from both health service and societal perspectives for individuals aged 50 years and older (Turner et al., 2006; Aballéa et al., 2007). Another study developed mathematical models to assess the cost benefit of sharing pediatric and adult flu vaccines (Chen, 2017). Influenza vaccination can also benefit the younger population. Scholars estimate the clinical impact of introducing childhood influenza vaccine in England and Wales (Pitman, White & Sculpher, 2012). They further develop a dynamic transmission model to assess the cost-effectiveness for the new vaccination policy in the same region (Pitman, Nagy & Sculpher, 2013). Other scholars analyzed the impact of immunizing school-age children to economic loses and domestic transmission (Neuzil, Hohlbein & Zhu, 2002; Yoo et al., 2013).

The epidemic model provides an opportunity to understand how diseases spread in the population. The initial study by Kermack and McKendrick classified the population as the susceptible, infected, and recovered (SIR) categories, and used mathematical expressions for describing the evolution of each compartment (Kermack & McKendrick, 1927). Extending from this foundation, Brogger incorporated vaccine recipients with the disease model for analyzing tuberculosis transmission (Brogger, 1965). Other studies developed disease models to investigate vaccination impacts on different diseases (Hethcote & Waltman, 1973; Revelle, Lynn & Feldmann, 1967; Waaler, Geser & Anderson, 1962).

Recently, attention has turned towards understanding the effectiveness of a vaccination strategy by use of disease modeling. Ball, Mollison & Scalia-Tomba (1997) developed an SIR model with a stratified population in households to obtain the optimal threshold for controlling an epidemic. Becker and Starczak assessed the post-vaccination reproduction number in a stochastic SIR framework and suggested minimal vaccination coverage to prevent disease transmission (Becker & Starczak, 1997). Another study applied mathematical programming models to determine vaccine allocation decisions for multiple regions against influenzas (Tanner, Sattenspiel & Ntaimo, 2008). Muller used a SIRS (susceptible-infected-recovered-susceptible) epidemic model to obtain the optimal vaccination coverage for different age groups (Müller, 1997). Hill and Longini proposed a general framework to obtain optimal vaccination strategies to apply to several types of epidemic models, including SIR models, SEIR (susceptible-exposed-infected-recovered) models, and SIS (susceptible-infected-susceptible) models (Hill & Longini, 2003).

Yarmand et al. developed a simulation model to capture the epidemic dynamics in different regions and formulated the vaccine allocation problem as two-stage stochastic programming. Using the 2-SLP formulation, they estimated the value of the stochastic solution and the expected value of perfect information. The results showed that the proposed two-phase vaccination policy potentially resulted in a lower attack rate and a considerable saving in vaccine production and administration costs (Yarmand et al., 2014).

The time-series model can be utilized to understand how the disease transmitted in population over time. Sophisticated approaches focused on periodic outbreaks by integrating mathematical formulations with actual epidemical data. For example, Finkenstädt et al. developed models to estimate weekly transmission rates and unreported cases using surveillance data for measles in England and Wales. Their computational results were coherent with the actual transmission pattern and capable of explaining the biennial cycle of measles outbreaks (Finkenstädt & Grenfell, 2000). Another study applied time-series regression model to investigate the effectiveness of mass vaccination for controlling influenzas in Taiwan (Wu et al., 2014).

From a retrospective review of the immunization strategy in 2009, simply ordering vaccines is insufficient for controlling influenza, as well as the effectiveness of timely implementing vaccination program is rarely discussed by literature. The present study aims to address these challenges. We compare the previous vaccine coverage and the expanding program to order double vaccine doses to cover people 13–18 and 50–64 years old. The analysis includes various vaccine policies of vaccination timing and campaign size using the epidemic data collected from both 2009 and the recent years. The remaining sections of this paper are organized as follows.‘Materials and Methods’ describes the stochastic disease model, parameter setting, and assumptions. ‘Results’ presents the result of the simulation experiment. The final section is the discussion and conclusions.

## Materials and Methods

### Disease model

The discrete-time compartmental model considers the transition rate from one population group to another by use of difference equations to determine the epidemic status over time. In the simulation study, we developed and applied the model to evaluate the effectiveness of vaccine policies. The stochastic SIR model was implemented on the R language version 3.4.2 to sample new infections and then compute the number of individuals in each compartment (R Core Team, 2017). The foundation of the compartmental model may refer to (Brauer & Castillo-Chávez, 2001; Daley & Gani, 1999). Our methodology is closely related to the pioneer study of applying disease models to understand measles epidemics in the UK (Finkenstädt & Grenfell, 2000). As opposed to the prior work we considered more details on vaccination activities, which include multiple vaccine types and their characteristics, vaccination timing, and recipient arrival frequencies. Additionally, we segregated the population by age and county according to the demographic data in Taiwan. The time horizon was one year and time unit is weekly in our model. We assumed that the population was closed, and there was no traveling or migration within the study period. However, this assumption can be relaxed by adding the birth and mortality rates to the compartmental model accordingly.

Let *N*_*a*,*j*,*t*_ be the total population of age group *a* at location *j* at time period *t*, *γ* be the recovery rate, and *e* be the vaccine efficacy. The variables of *S*_*a*,*j*,*t*_, *I*_*a*,*j*,*t*_, Δ*I*_*a*,*j*,*t*_, *R*_*a*,*j*,*t*_ and *V*_*a*,*j*,*t*_ refer to the numbers of susceptible, infected, new infected, recovered and vaccinated individuals of age group *a* at location *j* at time period *t*, respectively. The parameter of *β*_*t*_ represents the transmission rate at time period *t*. The population size of each compartment will be changed over time according to the following equations. The first equation defines the number of susceptible individuals, which is the number of susceptible individuals in the previous time period, subtracted from the new infected (Δ*I*_*t*−1_) and immunized individuals (*eV*_*a*,*j*,*t*−1_).

(1) \begin{eqnarray*}{S}_{a,j,t}={S}_{a,j,t-1}-\Delta {I}_{a,j,t-1}-e{V}_{a,j,t-1}.\end{eqnarray*}

The values of Δ*I*_*t*−1_ and *eV*_*a*,*j*,*t*−1_ are sampled from the *Poisson* distribution in the simulation experiment. The expected value of Δ*I*_*t*−1_ is defined by Eq. (3), and the expected value of immunized individuals is equal to the vaccine efficacy multiplying the maximal value of arrival population and vaccine availability.

Next, we explain the equation for determining the number of infected individuals. Given the recovered rate *γ* during a time period, the number of infected individuals at the end of the time period *t* is equal to the initial infected cases minus the new recovered cases plus the new infected cases:

(2) \begin{eqnarray*}{I}_{a,j,t}={I}_{a,j,t-1}-\gamma {I}_{a,j,t-1}+\Delta {I}_{a,j,t-1}.\end{eqnarray*}

Similarly, both *γI*_*a*,*j*,*t*−1_ and Δ*I*_*a*,*j*,*t*_ are sampled randomly from Poisson distributions using of the expected values of recovered rate multiplied by the last time period infections, and the new infected defined in Eq. (3), respectively.

Equation (3) defines the expected number of new infected individuals, which is the multiplication of transmission rate, susceptible individuals, and total infected individuals, and then divided by the population of age group *a*.

(3) \begin{eqnarray*}\Delta {I}_{a,j,t}={\beta }_{t-1}{S}_{a,j,t-1}\sum _{a\in A}{I}_{a,j,t-1}/\sum _{a\in A}{N}_{a,j,t-1}.\end{eqnarray*}

Finally, the number of recovered individuals is equal to the cumulative recovered individuals, new recovered individuals in the last time period, and immune individuals as follows.

(4) \begin{eqnarray*}{R}_{a,j,t}={R}_{a,j,t-1}+\gamma {I}_{a,j,t-1}+e{V}_{a,j,t-1}.\end{eqnarray*}

### The transmission rate

The ILI data were collected from the CDC in Taiwan for determining the weekly transmission rate (Centers for Disease Control, 2017a). The surveillance data only included reported cases without symptomatic ones. The epidemic scenarios in 2009 and regular season scenarios calculated in different ways stated as follows. In the new H1N1 pandemic scenario, we applied the reported ILIs in 2009 directly to determine the weekly transmission rate in 2009. In the regular epidemic scenario, the weekly transmission rate was estimated by use of the multi-year data of 2013–2015. The parameter value was obtained by the *least square method* to minimize the sum of the squared deviation between actual new infections and fitted value. The fitting result was performed well for the estimation during 2013 and 2015, where the correlation coefficient was close to one, and the sum square of errors was small. Table 1 displays the weekly transmission rates used in this study.

**Table 1:** The weekly transmission rate.

Year	Weekly transmission rates^a^
*Regular epidemic*	{0.953, 0.979, 0.946, 0.959, 0.972, 1.008, 0.954, 0.984, 1.046, 1.029, 1.004, 0.998, 1.172, 1.022, 1.042, 1.017, 1.067, 1.075, 1.078, 1.061, 1.089, 1.056, 1.042, 1.206, 1.108, 1.176, 1.166, 1.081, 1.307, 1.244, 1.238, 1.265, 1.326, 1.233, 0.985, 1.406, 1.385, 1.186, 1.125, 1.156, 1.158, 1.219, 1.06, 1.135, 1.207, 1.157, 1.109, 1.191, 1.193, 1.208, 1.273, 1.334}
*2009-like pandemic*	{0.912, 0.999, 0.984, 1.042, 1.001, 1.02, 1.061, 1.09, 0.958, 1.148, 1.129, 1.334, 1.213, 1.102, 0.934, 0.977, 0.944, 1.2, 1.151, 1.237, 1.201, 1.11, 1.135, 1.236, 1.317, 0.965, 0.955, 0.897, 1.157, 1.131, 1.206, 1.24, 1.101, 1.148, 1.273, 0.584, 2.462, 1.156, 1.389, 1.307, 1.352, 1.299, 1.121, 1.236, 1.248, 1.33, 1.279, 1.305, 1.173, 1.237, 1.246, 0.963}

**Notes.**

a The list of weekly transmission rates represents from week 23 in one year to week 22 in the next year.

The weekly transmission rate was either determined or estimated to provide the intensity of infection for the disease model simulation. If the value is greater than one, then the new infected cases will increase in the next time period. In 2013-2015, the transmission rates were continuously above one from the 31st week. While in 2009, an earlier epidemic began from the 26th week.

### Model validation

**Figure 2:**
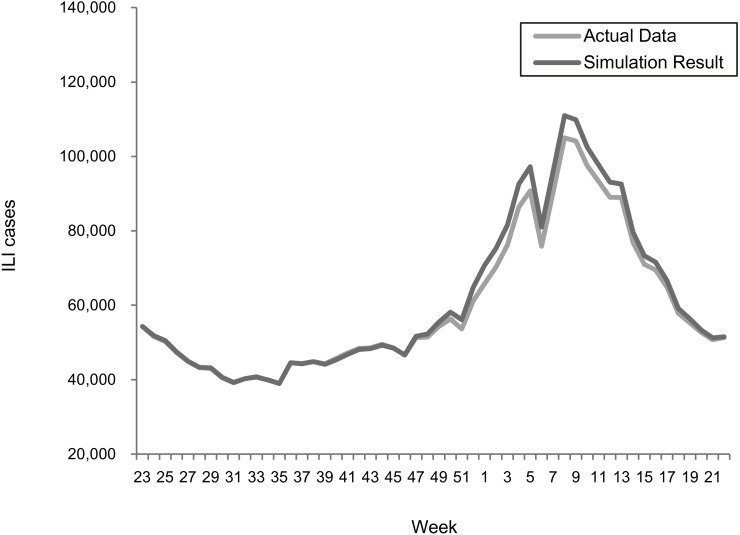
Comparing actual ILIs and simulation results in the scenario of the 2013–2015 seasons.

**Figure 3:**
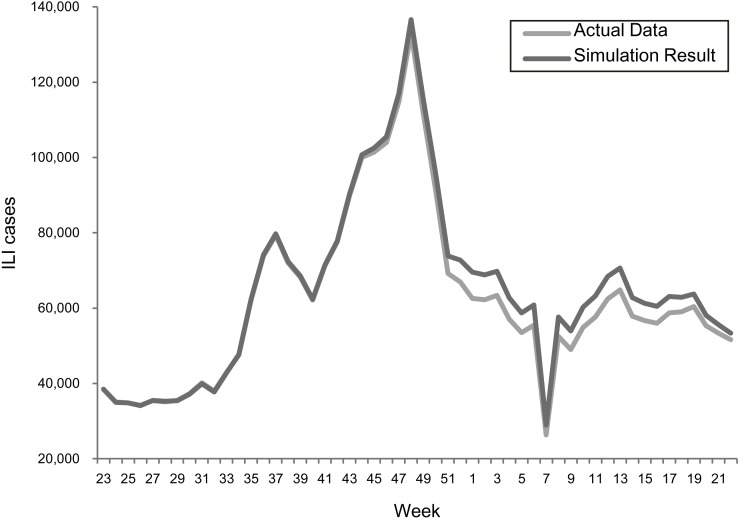
Comparing actual ILIs and simulation results in the scenario of the 2009 season.

The transmission rates were validated using a stochastic disease model to examine the forecasting accuracy for each epidemic scenario. Figures 2 and 3 summarize the comparisons between the predicted and actual infected cases in the regular epidemics and 2009-like pandemics, respectively.

Both results showed that the actual and simulated ILIs were very similar in the ways of pattern and scale. The sums of absolute error defined as —simulated infections—actual— actual are 3.0% and 3.6% for the 2013–2015 and 2009 scenarios, respectively. This confirmed the suitability of using the estimated transmission rates for predicting epidemic outcomes under different vaccination programs.

## Results

### Data and parameters

The vaccination rate used the empirical data of 35% for children ≤3 years old, 75% for 4–18 years old population, and 44% for the population 65 years old and above. The arrival rate for the population aged 50–64 is assumed as 35%. We assumed that the new infections and recipient arrivals were Poisson distributed with the parameter setting as follows. The weekly arrival percentage was determined by the actual vaccine coverage rates divided by the number of weeks during the vaccination period. Then, the mean of Poisson distribution used the weekly arrival percentage multiplied by the number of the target population. When vaccine inventory is in short, the distribution policy considers a fair fill rate across all locations. Population data were collected from the government, and stratified by six age groups of 0–3, 4–12, 13–18, 19–49, 50–64, and ≥65, and twenty-two counties (Department of Household Registration, 2017). There are two vaccine types applied to different age groups. The pediatric vaccine was for the children less than or equal to three years old, and the adult vaccine was for the remaining age groups. The time unit was weekly, and there were fifty-two time periods in a simulation run.

### Vaccination policies

The experiments were designed by referring to the current immunization program in Taiwan. Two levels of vaccination scales and four levels of campaign duration were considered to explore the effects of vaccination policies. Table 2 summarizes the studied policies. The first class of policies (from A-1 to A4) considered the vaccination scale implemented prior to 2016 that ordered 3 million doses of vaccine, and covered the population aged 0 to 12 and above 65 years old. Another set of policies (from B-1 to B-4) referred to the current implemented program of 6 million ordered doses with the target population of age 0 to 18 and above 50 years old. All vaccination policies were launched on week 40 (October first). Four scenarios on vaccination duration were investigated. The longest durations (A-1) lasted 4 months followed by the A-2 policy in 3 months, the A-3 policy in 2 months, and the shortest policy of A-4 in 1 month. A similar setting was applied for the policies of B-1 to B-4.

**Table 2:** Vaccine doses, target population and vaccination duration of potential policies.

Policy	Vaccine doses	Target population	Vaccination duration
Policy A-1	Pediatric:0.3million, Adult:2.7million	0∼12 and 65 + years old	Week40–Week 4 in the next year
Policy A-2	Pediatric:0.3million, Adult:2.7 million	0∼12 and 65 + years old	Week40–Week 52
Policy A-3	Pediatric:0.3million, Adult:2.7 million	0∼12 and 65 + years old	Week40–Week 48
Policy A-4	Pediatric:0.3million, Adult:2.7 million	0∼12 and 65 + years old	Week40–Week 44
Policy B-1	Pediatric:0.3million, Adult:5.7million	0∼18 and 50 + years old	Week40–Week 4 in the next year
Policy B-2	Pediatric:0.3million, Adult:5.7million	0∼18 and 50 + years old	Week 40–Week 52
Policy B-3	Pediatric:0.3million, Adult:5.7million	0∼18 and 50 + years old	Week 40–Week 48
Policy B-4	Pediatric:0.3million, Adult:5.7million	0∼18 and 50 + years old	Week 40–Week 44

### Epidemic scenarios

The effectiveness of vaccination policies was evaluated under various epidemic scenarios. The first scenario (regular epidemic) used the estimated the weekly transmission rates of 2013–2015. Furthermore, vaccine efficacy was set as 59% according to the summary of the literature review (Osterholm et al., 2012). The second scenario (2009-like pandemic) considered the irregular transmission pattern by using the actual transmission rate in each week in 2009. The transmission rate in each county was in similar due to the high population density and frequent domestic travels in Taiwan. Thus, our analysis used a unify transmission rate for all locations and assumed that the infected population could transmit diseases to the susceptible population within the same county. The vaccine efficacy was also reviewed according to the laboratory experiment for the monovalent vaccines reported in the literature. The simulation parameters for different vaccination policies under regular epidemic and 2009-like pandemic scenarios are summarized in Table 3.

**Table 3:** Epidemic scenarios and parameter values for the simulation experiment.

Parameter	Values	Sources
Population	23,499,404	Department of Household Registration (2017)
Age groups	0–3 yrs, 4–12 yrs, 13–18 yrs, 19–49 yrs, 50–64 yrs, and 65 + yrs	Assumed
Locations	22 counties	Department of Household Registration (2017)
Vaccination age groups	*A- policies*: 6m-3 yrs, 4–12 yrs, 13–18 yrs, 50–64 yrs, and 65 + yrs *B- policies*: 6m-3 yrs, 4–12 yrs, 13–18 yrs, and 65 + yrs	Taiwanese CDC
Recipient arrival rates	≤* 3 yrs*: 35% *4–18 yrs*: 75% *50–64 yrs*: 35% *65*+* yrs*: 44%	Taiwanese CDC
Vaccine ordering quantity	*A- policies*: 2.7 million doses of adult vaccine and 0.3 million doses of pediatric vaccine *B- policies*: 5.7 million doses of adult vaccine and 0.3 million doses of pediatric vaccine	Taiwanese CDC
Vaccine efficacy	*Regular epidemic scenario:*	
	59% (average)	Osterholm et al. (2012)
	50% (below average)	Osterholm et al. (2012)
	20% (low)	Assumed (10%–60% Centers for Disease Control and Prevention (2018))
	*2009-like pandemic scenario:*	
	69%	Osterholm et al. (2012)
Vaccination periods	October October–November October–December October–January	Assumed
Time period	52 weeks	
Transmission rates	Table 1	Estimated or calculated using of the data of reported ILIs Centers for Disease Control (2017a)

### Simulation results

The stochastic disease model obtained vaccination outcomes under different epidemic scenarios. We reported the simulation result in Tables 4 and 5 for the regular epidemic and the 2009-like epidemic, respectively. The average ILIs were calculated based on five replications of simulation. Also, the actual ILIs (in the first row of the tables) provided a baseline for evaluating vaccination policies. The cases averted in the A-1 to A-4 policies represented the decrease or increase of new infections by adjusting vaccination durations. While the cases averted in the B-1 to B-4 policies implied the effects of augmenting the vaccine coverage from the previous season of three million doses to six million doses.

**Table 4:** Annual ILIs and cases averted in the regular epidemic scenario.

	Policy	Annual ILIs		Cases averted
*Reported cases during the 2013–2015 seasons*		3,098,632		N/A
*Simulation results*		*Average*	*Standard deviation*	
	Policy A-1	3,488,246	20,616	−389,614^a^
	Policy A-2	3,188,295	17,898	−89,663^a^
	Policy A-3	2,972,484	19,676	126,148^a^
	Policy A-4	2,745,710	43,607	352,922^a^
	Policy B-1	2,163,610	14,191	1,324,635^b^
	Policy B-2	1,912,309	12,920	1,275,985^b^
	Policy B-3	1,685,328	31,474	1,287,156^b^
	Policy B-4	1,466,097	19,774	1,279,613^b^

**Notes.**

a Cases averted are the reported cases minus the annual ILIs of the A- policies.

b Cases averted are the annual ILIs of the A- policies minus the annual ILIs of the B- policies, respectively.

**Table 5:** Annual ILIs and cases averted in the 2009-like epidemic scenario.

		Annual ILIs		Cases averted
*Reported cases in the 2009 season*		3,234,303		N/A
*Simulation results*		*Average*	*Standard deviation*	
	Policy A-1	3,604,619	26,850	−370,316^a^
	Policy A-2	3,368,654	13,862	−134,351^a^
	Policy A-3	3,124,538	23,631	109,765^a^
	Policy A-4	2,873,130	27,691	361,173^a^
	Policy B-1	2,402,780	14,114	1,201,839^b^
	Policy B-2	2,188,831	30,575	1,179,823^b^
	Policy B-3	1,932,464	14,394	1,192,074^b^
	Policy B-4	1,684,808	11,037	1,188,321^b^

**Notes.**

a Cases averted are the reported cases minus the annual ILIs of the A- policies.

b Cases averted are the annual ILIs of the A- policies minus the annual ILIs of the B- policies, respectively.

In Table 4, the numbers of new infections decreased when shortening the vaccination duration (A-1 > A-2 > A-3 > A-4, and B-1 > B-2 > B-3 > B-4). Vaccination programs during the 2013–2015 seasons were completed in three months. Thus, the policies shorter than three months (i.e., A-3 and A-4) obtained more positive cases averted. Policies with the shortest duration (A-4 and B-4) reduced infections from the longest duration policies (A-1 and B-1) by 742,536 reduced new infected cases for the three million doses policy and 697,513 reduced new infected cases for the six million doses policy. When doubling the vaccination scale, the B policies averted approximately 1.27 million more cases of infection than the corresponding polices. Policy B-4 obtained the best result among the eight policies.

**Figure 4:**
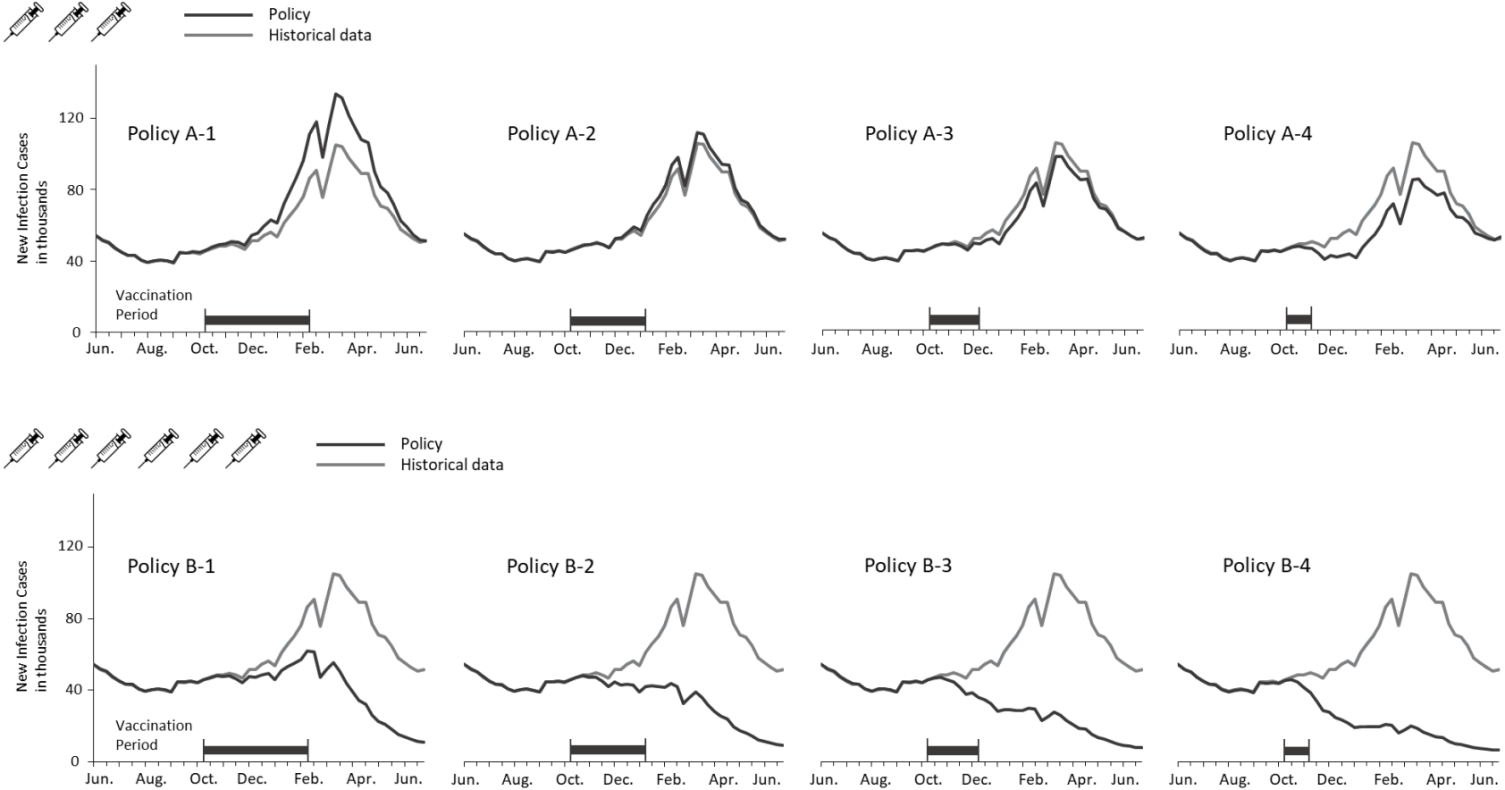
Comparing weekly ILI cases between the actual and vaccination policies in the regular epidemic scenario.

Figure 4 depicts the curves of weekly infections for the actual and vaccination policies. The policy A-2 obtained the closest estimation because the settings of coverage population and vaccination duration are identical with the actual situation. A considerable decline in the B policies indicated the effect of adding the three million doses to the vaccination campaign. Additionally, shortening the vaccination period lowered the peaks of transmission in both cases of coverage population. It is notable that no epidemic peaks occurred when vaccinating six million doses within three months (i.e., B-2, B-3, and B4 policies).

Table 5 displays the vaccination policies’ performances in the 2009-like epidemic scenario. The overall infections were greater than the regular epidemic scenario due to the higher transmission rates in 2009. The number of new infections reduced when shortening the vaccination period (109,765 for policy A-3 and 361,173 for policy A4), while policies with long vaccination period led to a negative number of cases averted (−370,316 for policy A-1 and −134,351 for policy A-2). Only augmenting vaccination doses obtained a lessening effect on the cases averted compared with the “regular epidemic” scenario, in which cases averted are 1,201,839∼1,188,321 the in the 2009-like epidemic scenario versus 1,324,635∼1,279,613 in the regular epidemic scenario. The explanation is that the first outbreak in 2009 occurred before the vaccines were available, and therefore, the immunization program could only avert the infections during the second wave in December.

**Figure 5:**
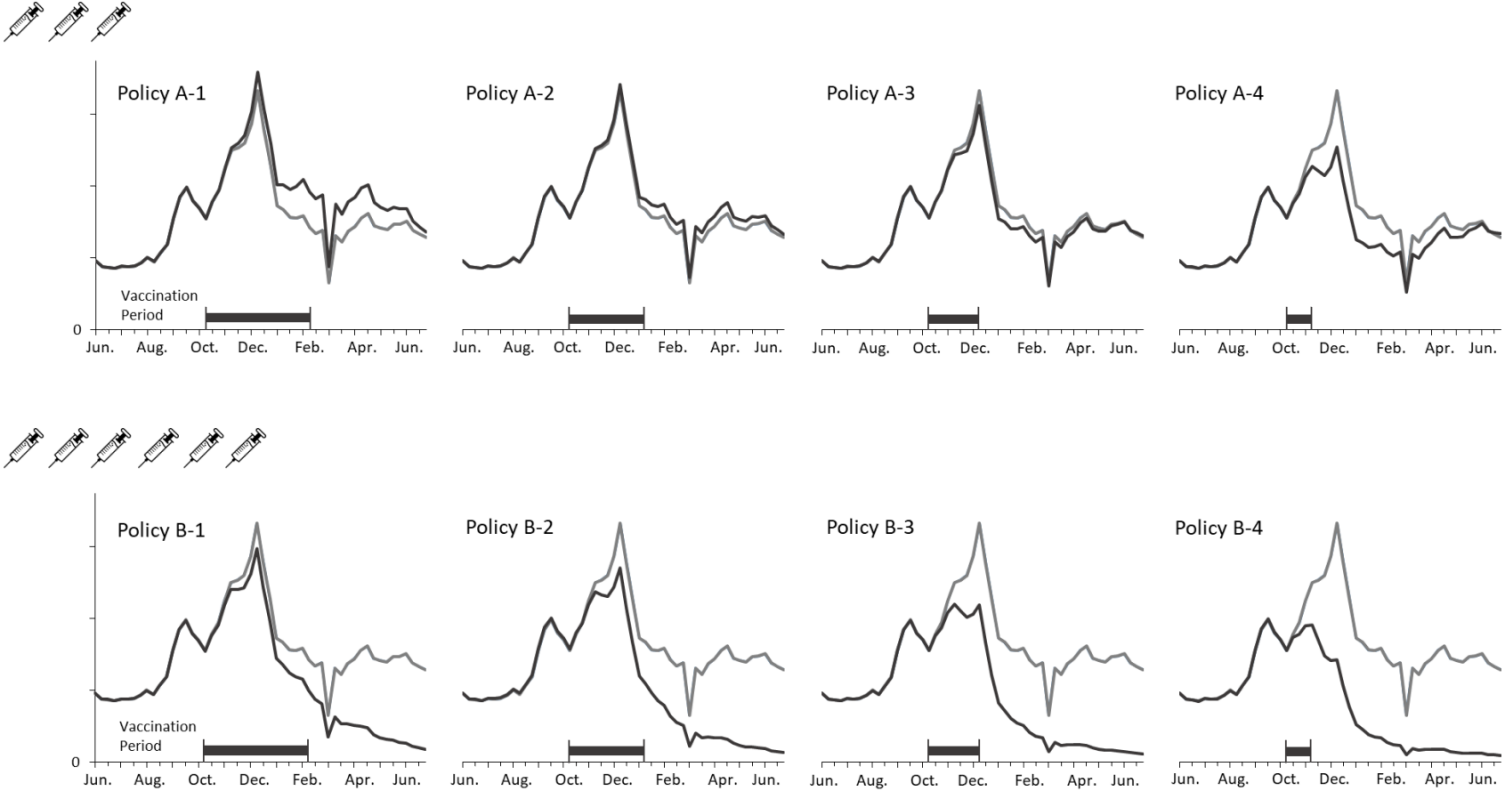
Annual ILIs and cases averted in the 2009-like epidemic scenario.

Figure 5 presents new infections each week to understand the new H1N1 outbreak concerning the immunization outcomes of vaccination policies. The first epidemic in Taiwan was observed in September followed by another larger outbreak in December. Vaccines were available for the target population after the first outbreak. Consequently, all vaccination policies did not affect the transmission prior to October. Both shortening the vaccination period and adding vaccine doses mitigated the total infections, but were unable to aviod major outbreaks in December until the vaccine period was reduced by one month. A significant reduction in the peak level during the outbreak in December was observed for the A-4, B-3 and B-4 policies. Also, administering 3 million doses in one month (A-4) obtained a lower peak level than vaccinating 6 million doses in 3 or 4 months (B-1 and B-2). These results imply that implementing an immediate vaccination activity during the epidemic can control the diseases effectively.

### Sensitivity analysis on the vaccination efficacy

**Figure 6:**
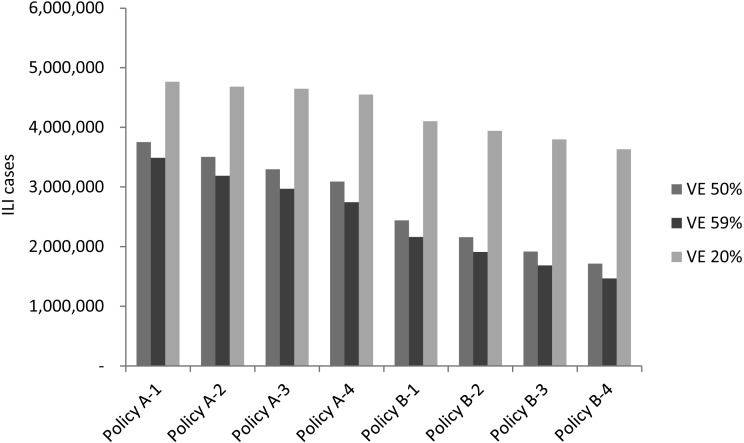
The simulation result of new infected cases when vaccine efficacy is 20%, 50% or 59%.

Vaccine efficacy fluctuation is usually associated with the drifting or recirculating of influenza viruses. In addition to investigating different epidemic scenarios, an analysis of both regular epidemic and 2009 pandemic scenarios with average vaccine efficacy (59%), below-average vaccine efficacy (50%), and the lowest vaccine efficacy (20%) was conducted to assess the effect that vaccine efficacy had on vaccination policies. Figure 6 displays the simulation result of average new infected cases for the three vaccine efficacy levels. As one can expect, all vaccination policies obtained more infections when vaccine efficacy decreased. For example, infected cases were increased between 250,000 to 350,000 for each policy if vaccine efficacy decreased from 59 to 50%. A larger scale of increasing on new infections observed when vaccine efficacy reduced to 20%.

**Figure 7:**
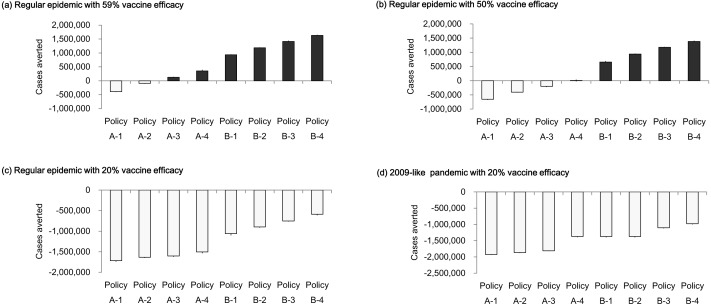
Cases averted by vaccination policies under different vaccine efficacy levels. (A) Regular epidemic with 59% vaccine efficacy; (B) regular epidemic with 50% vaccine efficacy; (C) regular epidemic with 20% vaccine efficacy; (D) 2009-like pandemic with 20% vaccine efficacy.

Figure 7 displays cases averted by vaccine policies under various vaccine efficacy levels. In the regular epidemic scenarios, when vaccine efficacy was below the average value (50% vaccine efficacy), most A- policies obtained negative numbers of cases averted except for the policy that completed vaccination in one month (Fig. 7B). Compared to the situation when vaccine efficacy performed regularly (59% vaccine efficacy), the cases averted became positive when the vaccine period was shorter than three months (Fig. 7A). The policies of providing double vaccine doses (B1-B4 policies) averted more than 656,045 new infections (about 21% reduction from base case) if the vaccine efficacy is equal to or above 50%. Figure 7C reports cases averted when vaccine efficacy is 20%. All vaccination polices obtained a more close performance (−1,719,179∼−587,740 averted cases) compare with the simulation results using higher vaccine efficacy. In the presence of regular (59%) vaccine efficacy, potential infections can be averted by either adding vaccination doses to the population or reducing vaccination period. When the vaccine efficacy is 50%, the number of infections can be reduced only by adding vaccination doses. There is no chance to reduce infections if vaccine efficacy is as low as 20%. We further analyze the low vaccine efficacy effect in the 2009-like pandemic scenario (Fig. 7D). Either reducing vaccination duration or increasing vaccination doses cannot reduce the number of cases averted.

## Discussion

In the regular epidemic scenario, the policies of covering 3 million people (referring to the programs implemented in Taiwan prior 2016) cannot prevent influenza outbreaks effectively (A-1, A-2, A-3, and A-4 policies in Fig. 4). However, the total infections are reduced if vaccination activities can be completed early. On the other hand, the number of cases averted can be lost by 110% (decreasing from 352,922 to -389,614) if vaccination timing is not early enough. When considering a double campaign size of 6 million doses (the current program in Taiwan), there are no epidemics if vaccination activities are completed within 3 months (B-2, B-3, and B-4 policies in Fig. 4). A small-scale outbreak still occurs for the policy with longest vaccination duration. This emphasizes the importance of quantity and timing in designing a vaccination program against influenzas.

In the 2009-like pandemic scenario, the early outbreak in September restricts the effectiveness of using government-funded vaccines. However, both increasing vaccine doses and reducing the program’s duration can mitigate the second-wave outbreak in December. The cases averted can be lost by 103% (decreasing from 361,173 to -370,316 for the 3-million doses polices) if vaccines are given to target population too late. The key to eradicating the recirculated outbreak in December is to cover a broader population sooner (see B-4 policy in Fig. 5).

The uncertain vaccine efficacy is an important consideration for planning a vaccination program. Sensitivity analysis indicates that the policies of doubling vaccination doses can still mitigate the amplitude of infections although the vaccine efficacy is at a below-average level. Neither increasing vaccination doses nor reducing vaccination period reduce infections when vaccine efficacy is as low as 20%.

## Conclusions

This study represents the first attempt to explicitly analyze the ILI time-series data during the 2009 H1N1 pandemic and the recent epidemics in Taiwan. A stochastic compartmental model is developed to simulate various epidemical scenarios. The simulation result is consistent with the actual ILIs reported by the government. Additionally, we apply the model to assess current and potential vaccination policies. Sensitivity analysis examines how different vaccine efficacies affected the vaccination performances in terms of infections and cases averted.

Our findings inform recommendations for public health policies. For example, vaccination programs may be able to deter epidemics not only by increasing coverage population but also by reducing the timeframe of vaccination activity. Furthermore, the policymaker should consider the uncertainty of vaccine efficacy when making decisions. According to the sensitivity analysis, the policies of shortening vaccination timeframe reduced infections for regular vaccine efficacy, but not for the case of below average. Both increasing vaccine doses and reducing vaccination timeframe fails to avert cases if vaccine efficacy is at an extremely low level of 20%.

The limitation of the present study state as follows. The transmission rate can be dissimilar for individuals with different age groups, while our analysis uses a mixed transmission rate across all age group population. Because of the availability of the ILI data limits us to obtain transmission rates for each specific population group. Future study may integrate the population-based prospective survey as an alternative source of the age-specific mixing data (Mossong et al., 2008). Finally, our analysis did not consider asymptomatic cases. We believe that including asymptomatic cases into the simulation model would increase the scale of infections, but not affect the conclusion we made. Without loss of generality, our model can be adjusted easily to adapt to these considerations when more fine data are available in the future.

##  Supplemental Information

10.7717/peerj.6340/supp-1 File S1 Reported ILIs, transmission rates, and simulation results

10.7717/peerj.6340/supp-2 File S2 The source code of the stochastic compartmental model for simulating influenza epidemics in Taiwan

## Additional Information and Declarations

### Data Availability

The following information was supplied regarding data availability: The raw measurements are available in the Supplementary Files.

## References

[ref-1] Aballéa S, Chancellor J, Martin M, Wutzler P, Carrat F, Gasparini R, Toniolo-Neto J, Drummond M, Weinstein M (2007). The cost-effectiveness of influenza vaccination for people aged 50 to 64 years: an international model. Value Health.

[ref-2] Ball F, Mollison D, Scalia-Tomba G (1997). Epidemics with two levels of mixing. The Annals of Applied Probability.

[ref-3] Becker NG, Starczak DN (1997). Optimal vaccination strategies for a community of households. Mathematical Biosciences.

[ref-4] Brauer F, Castillo-Chávez C (2001). Mathematical models in population biology and epidemiology. Texts in applied mathematics.

[ref-5] Brogger S (1965). Systems analysis in tuberculosis control: a model. American Review of Respiratory Disease.

[ref-6] Centers for Disease Control, Taiwan (2017a). Taiwan national infectious disease statistics system. https://nidss.cdc.gov.tw/en/.

[ref-7] Centers for Disease Control, Taiwan (2017b). Taiwan’s response to the H1N1 influenza. http://www.cdc.gov.tw/uploads/files/4c6e5d73-12a1-4b7a-b9a1-486c0c118629.pdf.

[ref-8] Centers for Disease Control, Taiwan (2018). Influenza vaccine. https://www.cdc.gov.tw/uploads/files/201803/a33bd88f-b6ef-46ae-87f6-02cb4fe27eac.pdf.

[ref-9] Centers for Disease Control and Prevention (2018). Seasonal influenza vaccine effectiveness, 2004–2018. https://www.cdc.gov/flu/professionals/vaccination/effectiveness-studies.htm.

[ref-10] Chen SI (2017). Economic benefits of sharing and redistributing influenza vaccines when shortages occurred. PLOS ONE.

[ref-11] Chuang J, Huang AS, Huang W, Liu M, Chou J, Chang F, Chiu WT (2012). Nationwide surveillance of influenza during the pandemic (2009–10) and post-pandemic (2010–11) periods in Taiwan. PLOS ONE.

[ref-12] Daley DJ, Gani J (1999). Epidemic modeling: an introduction.

[ref-13] Department of Household Registration, Taiwan (2017). Household registration statistics data. https://www.ris.gov.tw/app/en/3910.

[ref-14] Finkenstädt BF, Grenfell BT (2000). Time series modelling of childhood diseases: a dynamical systems approach. Journal of the Royal Statistical Society: Series C (Applied Statistics).

[ref-15] Hethcote HW, Waltman P (1973). Optimal vaccination schedules in a deterministic epidemic model. Mathematical Biosciences.

[ref-16] Hill AN, Longini IM (2003). The critical vaccination fraction for heterogeneous epidemic models. Mathematical Biosciences.

[ref-17] Kermack WO, McKendrick AG (1927). A contribution to the mathematical theory of epidemics. Proceedings: Mathematical, Physical and Engineering Sciences.

[ref-18] Maciosek MV, Solberg LI, Coffield AB, Edwards NM, Goodman MJ (2006). Influenza vaccination: health impact and cost effectiveness among adults aged 50 to 64 and 65 and older. American Journal of Preventive Medicine.

[ref-19] Mossong J, Hens N, Jit M, Beutels P, Auranen K, Mikolajczyk R, Massari M, Salmaso S, Tomba GS, Wallinga J, Heijne J, Sadkowska-Todys M, Rosinska M, Edmunds WJ (2008). Social contacts and mixing patterns relevant to the spread of infectious diseases. PLOS Medicine.

[ref-20] Müller J (1997). Optimal vaccination strategies—for whom?. Mathematical Biosciences.

[ref-21] Neuzil KM, Hohlbein C, Zhu Y (2002). Illness among schoolchildren during influenza season. Archives of Pediatrics and Adolescent Medicine.

[ref-22] Osterholm MT, Kelley NS, Sommer A, Belongia EA (2012). Efficacy and effectiveness of influenza vaccines: a systematic review and meta-analysis. The Lancet Infectious Diseases.

[ref-23] Pitman RJ, Nagy LD, Sculpher MJ (2013). Cost-effectiveness of childhood influenza vaccination in England and Wales: results from a dynamic transmission model. Vaccine.

[ref-24] Pitman RJ, White LJ, Sculpher M (2012). Estimating the clinical impact of introducing paediatric influenza vaccination in England and Wales. Vaccine.

[ref-25] Poland GA (2010). The 2009–2010 influenza pandemic: effects on pandemic and seasonal vaccine uptake and lessons learned for seasonal vaccination campaigns. Vaccine.

[ref-26] R Core Team (2017). https://www.R-project.org/.

[ref-27] Revelle CS, Lynn WR, Feldmann F (1967). Mathematical models for the economic allocation of tuberculosis control activities in developing nations 1, 2. American Review of Respiratory Disease.

[ref-28] Tanner MW, Sattenspiel L, Ntaimo L (2008). Finding optimal vaccination strategies under parameter uncertainty using stochastic programming. Mathematical Biosciences.

[ref-29] Turner DA, Wailoo AJ, Cooper NJ, Sutton AJ, Abrams KR, Nicholson KG (2006). The cost-effectiveness of influenza vaccination of healthy adults 50–64 years of age. Vaccine.

[ref-30] Waaler H, Geser A, Anderson S (1962). The use of mathematical models in the study of the epidemiology of tuberculosis. American Journal of Public Health.

[ref-31] World Health Organization (WHO) (2009). World now at the start of 2009 influenza pandemic. http://www.who.int/mediacentre/news/statements/2009/h1n1_pandemic_phase6_20090611/en/.

[ref-32] World Health Organization (WHO) (2018). WHO surveillance case definitions for ILI and SARI, case definitions for influenza surveillance. https://www.who.int/influenza/surveillance_monitoring/ili_sari_surveillance_case_definition/en/.

[ref-33] Wu UI, Wang JT, Chang SC, Chuang YC, Lin WR, Lu MC, Lu PL, Hu FC, Chuang JH, Chen YC, TIDSNet for pH1N1 vaccination (2014). Impacts of a mass vaccination campaign against pandemic H1N1 2009 influenza in Taiwan: a time-series regression analysis. International Journal of Infectious Diseases.

[ref-34] Yarmand H, Ivy JS, Denton B, Lloyd AL (2014). Optimal two-phase vaccine allocation to geographically different regions under uncertainty. European Journal of Operational Research.

[ref-35] Yoo B, Humiston SG, Szilagyi PG, Schaffer SJ, Long C, Kolasa M (2013). Cost effectiveness analysis of elementary school-located vaccination against influenza—results from a randomized controlled trial. Vaccine.

